# Evidence for Transcriptional Factor Dysregulation in the Dorsal Raphe Nucleus of Patients with Major Depressive Disorder

**DOI:** 10.3389/fnins.2012.00135

**Published:** 2012-10-18

**Authors:** Ilan A. Kerman, René Bernard, William E. Bunney, Edward G. Jones, Alan F. Schatzberg, Richard M. Myers, Jack D. Barchas, Huda Akil, Stanley J. Watson, Robert C. Thompson

**Affiliations:** ^1^Department of Psychiatry and Behavioral Neurobiology, University of Alabama at BirminghamBirmingham, AL, USA; ^2^Department of Experimental Neurology, Charité HospitalBerlin, Germany; ^3^Department of Psychiatry, University of CaliforniaIrvine, CA, USA; ^4^Center for Neuroscience, University of CaliforniaDavis, CA, USA; ^5^Stanford UniversityPalo Alto, CA, USA; ^6^HudsonAlpha Institute for BiotechnologyHuntsville, AL, USA; ^7^Weill School of Medicine, Cornell UniversityNew York, NY, USA; ^8^Molecular and Behavioral Neuroscience Institute, University of MichiganAnn Arbor, MI, USA; ^9^Department of Psychiatry, University of MichiganAnn Arbor, MI, USA

**Keywords:** serotonin, postmortem, gene expression, microarray, real-time PCR, laser capture microdissection, depression

## Abstract

Extensive evidence implicates dysfunction in serotonin (5-HT) signaling in the etiology of major depressive disorder (MDD). Dorsal raphe nucleus (DR) is a major source of serotonin in the brain, and previous studies have reported within it alterations in 5-HT-related gene expression, protein levels, receptor binding, and morphological organization in mood disorders. In the present study, we utilized *in situ* hybridization-guided laser capture microdissection to harvest tissue samples from the middle-caudal subregion of the human DR post-mortem from MDD patients and from psychiatrically normal comparison subjects. Extracted RNA was prepared for gene expression profiling, and subsequent confirmation of select targets with quantitative real-time PCR. Our data indicate expression changes in functional gene families that regulate: (1) cellular stress and energy balance, (2) intracellular signaling and transcriptional regulation, and (3) cell proliferation and connectivity. The greatest changes in expression were observed among transcriptional regulators, including downregulation in the expression of TOB1, EGR1, and NR4A2 and their downstream targets. Previous studies have implicated these gene products in the regulation of functional domains impacted by MDD, including cognitive function, affective regulation, and emotional memory formation. These observations indicate altered function of several transcriptional regulators and their downstream targets, which may lead to the dysregulation of multiple cellular functions that contribute to the pathophysiology of MDD. Future studies will require single cell analyses in the DR to determine potential impact of these changes on its cellular functions and related circuits.

## Introduction

Major depressive disorder (MDD) is among the most debilitating neuropsychiatric illnesses. With an estimated lifetime prevalence of 16%, MDD is characterized by a high burden of morbidity caused by co-occurrence of physical ailments together with a high level of mortality due to suicide (Carney et al., 1993; Frank and Thase, 1999; Penninx et al., 2001; Kessler et al., 2003; Brown et al., 2004; Taylor and MacQueen, 2006). Despite increased public awareness and enormous research efforts, the etiology and pathophysiology of MDD remain poorly understood, and few fully effective treatments are available. The most compelling theory of the etiology of MDD has been the monoaminergic theory of depression, which posits a deficit in the concentration of monoaminergic neurotransmitters such as serotonin (5-HT) in the brains of depressed patients (Bunney and Davis, 1965; Schildkraut, 1965; Coppen, 1968; Lapin and Oxenkrug, 1969; Hirschfeld, 2000; Leonard, 2000). Consistent with this theory, first-line treatments for MDD include medications that increase absolute concentrations of monoaminergic neurotransmitters at the synapse, such as selective 5-HT reuptake inhibitors (SSRIs) or tricyclic antidepressants (Leonard, 2000; Blier, 2001). The theory also involves an assumption that, conversely, depletion of monoaminergic transmitters can induce a depressive state (Goodwin and Bunney, 1971; Neumeister, 2003).

The single largest source of serotonergic innervation of the forebrain is the dorsal raphe nucleus (DR; Azmitia, 1999). Via its dense innervation of forebrain limbic and cortical regions, the DR regulates affect, cognition, reward, and homeostasis, all of which are disturbed in MDD (Lowry et al., 2008). Previous studies have documented extensive alterations in the serotonergic system in the depressed brain, including alterations in 5-HT receptor binding, gene expression, protein levels, and reuptake transporter function in both the forebrain and the brainstem (Arango et al., 1996a,b, 2001, 2002; Klimek et al., 1997; Underwood et al., 1999; Zhu et al., 1999; Ordway et al., 2003; Boldrini et al., 2005, 2008; Bach-Mizrachi et al., 2006, 2008; Matthews and Harrison, 2012); these data suggest a disruption of 5-HT circuits both at the level of the cell body as well as the terminal. Consistent with this notion, a number of studies have documented gene expression alterations in the DR in depression (Arango et al., 2001; Boldrini et al., 2005; Bach-Mizrachi et al., 2006, 2008; Goswami et al., 2010).

All of these previous studies were hypothesis driven and focused solely on expression of genes that directly regulate 5-HT neurotransmission or 5-HT receptor signaling. Because MDD is a multifactorial and is likely a polygenic disorder, a dysregulated 5-HT pharmacologic state alone cannot account for all DR abnormalities. Therefore, the current study utilized an unbiased microarray-based approach to identify novel expression alterations of genes in the DR in MDD in postmortem human tissue. Our data indicate alterations in the expression of multiple transcriptional regulators that have the potential to impact diverse cellular functions within the DR and contribute to the etiology of MDD.

## Materials and Methods

### Subjects

Acquisition of postmortem human brain samples were conducted at the University of California at Irvine Brain Bank. Tissue processing, and procedures for microarray experiments have been previously described (Evans et al., 2004; Tomita et al., 2004; Choudary et al., 2005; Bernard et al., 2011). Brainstem tissue blocks were collected from depressed subjects (*n* = 13; antemortem diagnoses of MDD) and from non-psychiatric controls (C; *n* = 8; Table 1). Because extensive agonal stress and low tissue pH dramatically impact profiles of gene expression in the brain (Li et al., 2004; Tomita et al., 2004; Atz et al., 2007), blocks were collected only from subjects that experienced sudden deaths (agonal factor score of 0), and only samples from brains with pH > 6.6 were included in further processing (Table 1).

**Table 1:** **Demographic and clinical characteristics of subjects included in the study**.

Diagnosis	Subject	Race	Age	Sex	PMI (hours)	Agonal factor	Brain PH	Cause of death	SSRI at time of death
Control	2169	Caucasian	18	M	22	0	6.97	Accident	No
Control	2292	Caucasian	55	M	15	0	6.89	Sudden med. cond.	No
Control	2805	Caucasian	45	M	21	0	6.86	Sudden med. cond.	No
Control	3228	Pacific Islander	39	M	18.15	0	6.81	Sudden med. cond.	No
Control	3516	Caucasian	41	M	22.5	0	7.01	Sudden med. cond.	No
Control	3519	African American	65	M	13.5	0	6.88	Sudden med. cond.	No
Control	3520	Caucasian	74	F	18.5	0	7.21	Sudden med. cond.	No
Control	3588	Caucasian	56	M	24.5	0	6.98	Sudden med. cond.	No
MDD	2208	Caucasian	72	F	21	0	7.13	Suicide	No
MDD	2267	Caucasian	19	M	18	0	7.11	Suicide	No
MDD	3064	Caucasian	46	M	27	0	6.91	Sudden med. cond.	No
MDD	3169	Caucasian	35	M	24.75	0	7.04	Accident	No
MDD	3365	Caucasian	47	M	29	0	7.25	Suicide	No
MDD	3426	Caucasian	63	M	28.5	0	7.17	Sudden med. cond.	No
MDD	3031	Caucasian	49	M	27	0	7.19	Suicide	No
MDD	3398	Caucasian	80	F	15	0	6.68	Sudden med. cond.	No
MDD	2315	Caucasian	58	M	24	0	6.93	Suicide	Yes
MDD	2944	Caucasian	52	M	16	0	6.82	Sudden med. cond.	Yes
MDD	3071	Caucasian	49	M	31	0	7	Unknown	Yes
MDD	3168	Caucasian	39	M	27.5	0	6.79	Suicide	Yes
MDD	3481	Caucasian	66	M	32	0	7.05	Sudden med. cond.	Yes

*All subjects in the cohort were well matched for age, gender, race, post-mortem interval, agonal factor, and pH. F, female; M, male; MDD, major depressive disorder; cond., condition; PMI, post-mortem interval; SSRI, selective serotonin reuptake inhibitor*.

### Tissue processing

Tissue blocks were sectioned on a cryostat in a coronal plane to a thickness of 10 μm and immediately thaw mounted onto SuperFrost glass slides (Fisher Scientific, Pittsburgh, PA, USA). Each slide contained one section, and slides were stored at −80°C. Every 50th slide was then processed for radioactive *in situ* hybridization (ISH) to detect the distribution of serotonin transporter (SERT) mRNA as previously described (Lopez-Figueroa et al., 2004; Bernard et al., 2009). Specificity of the ISH probe was validated using sense controls and tissue pretreatment with RNase (data not shown). Processed tissue sections were apposed to radiosensitive film (BioMax MR film, Kodak^1^) for 7 days to delineate the anatomical boundaries of the DR.

Slides processed for ISH were then stained with a modified Klüver–Barrera method, which included staining with cresyl violet and luxol fast blue to identify neurons and fibers of passage. Distribution of SERT ISH signal was used in combination with histologically stained slides to delineate the regions of interest and to facilitate anatomical alignment of the specimens. This approach ensured that we sampled from the same antero-posterior subregion of DR common among all subjects in our cohort.

SERT autoradiograms and histologically stained slides were digitized and used to guide laser capture microdissection (LCM) on adjacent unstained tissue sections. It was important to use unstained tissue for LCM so as to preserve RNA integrity for the downstream gene expression assays. At the same time, use of the histologically and neurochemically stained adjacent guide sections allowed precise anatomical delineation of DR and related brainstem nuclei (Bernard et al., 2009, 2011). Samples were harvested from the middle-caudal DR, with bilateral collection from three consecutive tissue sections at three levels separated by 500 μm (total of nine DR nuclei). Position of anatomical landmarks, including the fourth ventricle and medial longitudinal fasciculus, from histochemically stained images, and SERT ISH signal were visually projected onto sections used for LCM. Regions in unstained sections that corresponded to boundaries of the DR were then microdissected under a 4× objective using CapSure macrocaps (Applied Biosystems) as previously described (Bernard et al., 2009). We have previously validated neuroanatomical precision of this approach (Bernard et al., 2009).

### RNA isolation and amplification

RNA extraction and isolation were performed with the use of the PicoPure RNA Isolation Kit (Molecular Devices, Sunnyvale, CA, USA) according to manufacturer’s instructions, including DNase treatment. For each nucleus, RNA extracts from the three LCM caps from the same subject were combined before purification. RNA quantity and quality were evaluated using the 2100 BioAnalyzer (Agilent Technologies, Palo Alto, CA, USA). RNA quality and its suitability for downstream gene expression analyses were determined from BioAnalyzer electropherograms according to the method of Schoor et al. (2003). Accordingly, extracted RNA samples were considered acceptable for downstream gene expression analyses if they met the following criteria: (1) <65% of the total area under the electropherogram curve was between the end of the marker peak and the beginning of the 18S rRNA peak, and (2) >4% of the total area was under the 28S rRNA peak (Schoor et al., 2003; Bernard et al., 2009). All of our samples met these exclusion criteria and were included in further analyses and processing.

RNA samples were subjected to two rounds of amplification (RiboAmp OA RNA Kit, Molecular Devices) and subsequent biotin labeling (Perkin Elmer, Waltham, MA, USA) according to manufacturers’ instructions. A portion of the second strand cDNA synthesis reaction product (i.e., amplified double-stranded cDNA), generated from RNA following *in vitro* transcription at the end of the first round of amplification was set aside for quantitative real-time PCR (qPCR; see below). After two amplification rounds, 15 μg of biotinylated amplified RNA from each DR sample were then hybridized to HG-U133 Plus 2.0 arrays (Affymetrix, Santa Clara, CA, USA) per manufacturer’s instructions.

### Gene expression microarrays

Affymetrix CEL files were analyzed with the use of the Robust Multi-Chip Average (RMA) and the Affymetrix Microarray Suite 5 calls (MAS5CALLS) algorithm. Affymetrix chip description files were replaced by custom probe set mapping files^2^ that independently reassigned all Affymetrix probe sets to an updated UniGene cluster (Dai et al., 2005). Log_2_-based microarray intensity values were used for statistical analyses.

Gene expression differences were evaluated by using Student’s *t*-tests and were considered to be significant if: (1) the *p*-values were ≤0.05, (2) expression was detected in ≥50% of arrays in C or MDD groups according to the MAS5CALLS algorithm, and (3) a gene’s log_2_ intensity value was ≥4.0 according to RMA algorithm. Fold change differences were calculated from group averages of microarray intensity values. Pearson’s correlations (two-tailed *p*-values) between microarray and qPCR results were computed using MS Excel 2010.

### Quantitative real-time PCR

We used SYBR Green chemistry for qPCR validation of microarray results. Genomic DNA and mRNA sequences were downloaded from NCBI Entrez Gene^3^, and Primer3 software (Rozen and Skaletsky, 2000) was used to design primers to: (1) anneal within 500 bp of the 3′ end, (2) generate a single amplicon 75–150 bp in size, and (3) amplify all splice variants for the gene of interest. For each amplicon, predicted secondary structure was minimized with the use of DNA Mfold (Zuker, 2003,^4^). Primer sequences are listed in Table 2. Primer performance was validated via serial dilutions and amplification efficiency testing by using human genomic DNA and/or amplified cDNA from human brainstem sections [source of input DNA did not impact amplification efficiency (data not shown)].

**Table 2:** **Primers used for qPCR**.

Gene name	Gene symbol	Accession number	Primers (forward, reverse)	*T*_m_ (°C)	Product size (bp)
Transducer of ERBB2, 1	TOB1	NM_005749	TTTTCATTTGCCAACCAAGC	60.98	115
			AAAATCAGCCATGTCCTTGC	60.08	
Nuclear receptor subfamily 4, group A, member 2	NR4A2	NM_173173	ACACCGTCCAACATTCCTTG	60.81	113
			TGCATGCAAGTTTTGTTTAGC	59.02	
CD69 molecule	CD69	NM_001781	AGACAGGTCCTTTTCGATGG	59.14	147
			TGCCACATCACATATTGCAC	58.95	
Early growth response 1	EGR1	NM_001964	TAGGCGGCGATTTTTGTATG	60.95	138
			TATCCCATGGGCAATAAAGC	59.76	
TCDD-inducible poly(ADP-ribose) polymerase	TIPARP	NM_015508	TTCCTCAGTTCAGAGAGGTAGC	57.82	139
			AAACATTACCAGGAGAGCTTGG	59.65	
Protein phosphatase 1, regulatory (inhibitor) subunit 3C	PPP1R3C	NM_003053	TGTGCACAACCATTGAGAGG	60.72	75
			TACTTTTCCACCATGGCACA	59.96	
Adrenomedullin	ADM	NM_001124	GTGAATGTCTCAGCGAGGTG	59.42	110
			TCGGTGTTTCCTTCTTCCAC	60.09	
CXCR7 chemokine (C-X-C motif) receptor 7	CXCR7	NM_020311	ATATTGTTTGGGAGGCATAGTG	58.03	75
			CAAAACTGAAGTCACGCTAACC	58.95	
Nuclear receptor subfamily 4, group A, member 3	NR4A3	NM_006981	TGTGAACATGCCTTCTGTGG	60.72	120
			GCAATGCTGTTAGAGGAGCAG	60.17	
Solute carrier family 19 (thiamine transporter), member 2	SLC19A2	NM_006996	TTGTGGCATACAACCTGAGC	59.72	97
			TGCACATTGAGTTCAGCATTC	59.86	
Eukaryotic translation elongation factor 1 alpha 1	EEF1A1	NM_001402	TTGACATGCAAGGAAGCAAG	59.99	138
			GTGCTCAAGCCACAGTTGTC	59.47	

*bp, base pairs; T_m_, melting temperature*.

Reactions were carried out in 96-well PCR plates (Bio-Rad, Hercules, CA, USA). Each well contained 5 μl of amplified double-stranded cDNA (50 pg/μl), which was set aside after the second strand cDNA synthesis performed following *in vitro* transcription at the end of first round of mRNA amplification (as described above). Concentration of amplified cDNA was quantified for each sample by using Quant-iT PicoGreen dsDNA Kit (Invitrogen, Carlsbad, CA, USA) according to manufacturer’s instructions. Each qPCR well also contained 5 μl of forward and reverse strand primers (final concentration 500 nM) and 10 μl of iQ SYBR Green Supermix (Bio-Rad).

A Bio-Rad iCycler (Bio-Rad) was utilized for amplifications, which were performed by using a touchdown PCR approach: denature at 95°C for 1 min 45 s, followed by ten cycles of (1) denaturing at 95°C for 15 s, (2) annealing at 65–60°C for 15 s, and (3) extension at 72°C for 15 s. Annealing temperature was decreased at each step of the cycle by 0.5°C from a maximum of 65°C to the final temperature of 60°C. This was followed by 45 cycles of (1) denaturing at 95°C for 15 s, (2) annealing at 60°C for 15 s, and (3) extension at 72°C for 15 s. Fluorescence was quantified after the extension step by using a FAM-490 or SYBR-488 detection protocol at a peak excitation wavelength of 490 nm and peak emission wavelength of 530 nm.

After amplification was completed, PCR products were denatured by sequential increases in temperature from 72 to 95°C in 0.5°C increments. At each step the temperature was held constant for 10 s, during which time fluorescence was quantified. The presence of specific amplification products was confirmed by the presence of a single peak on one of these melting curves, which were plotted as the negative derivative of fluorescence as a function of temperature. No template controls, in which DNA was replaced with distilled H_2_O, did not yield fluorescent signals (e.g., no amplification products).

All samples were amplified in triplicate, and an average cycle threshold (Ct) was calculated for each sample. Replicates that were ≥1 Ct away from the mean Ct were excluded; the mean Ct included only the remaining duplicates. Because input amount of amplified cDNA was equivalent across all samples, raw Ct values were inversely proportional to the levels of gene expression (Libus and Storchova, 2006). We chose this approach instead of normalization to housekeeping genes because of the potential for differential expression of such reference transcripts in disease versus control samples (Dheda et al., 2005; Wong and Medrano, 2005). A similar approach has recently been validated in which standardized DNA input amounts for qPCR were used (Bernard et al., 2009, 2011). The following formula was used to calculate relative fold changes: 2^−(Cta-Ctb)^ where Cta is cycle threshold in the MDD group and Ctb is cycle threshold in the C group.

## Results

### Organization of the dorsal raphe

**Figure 1:**
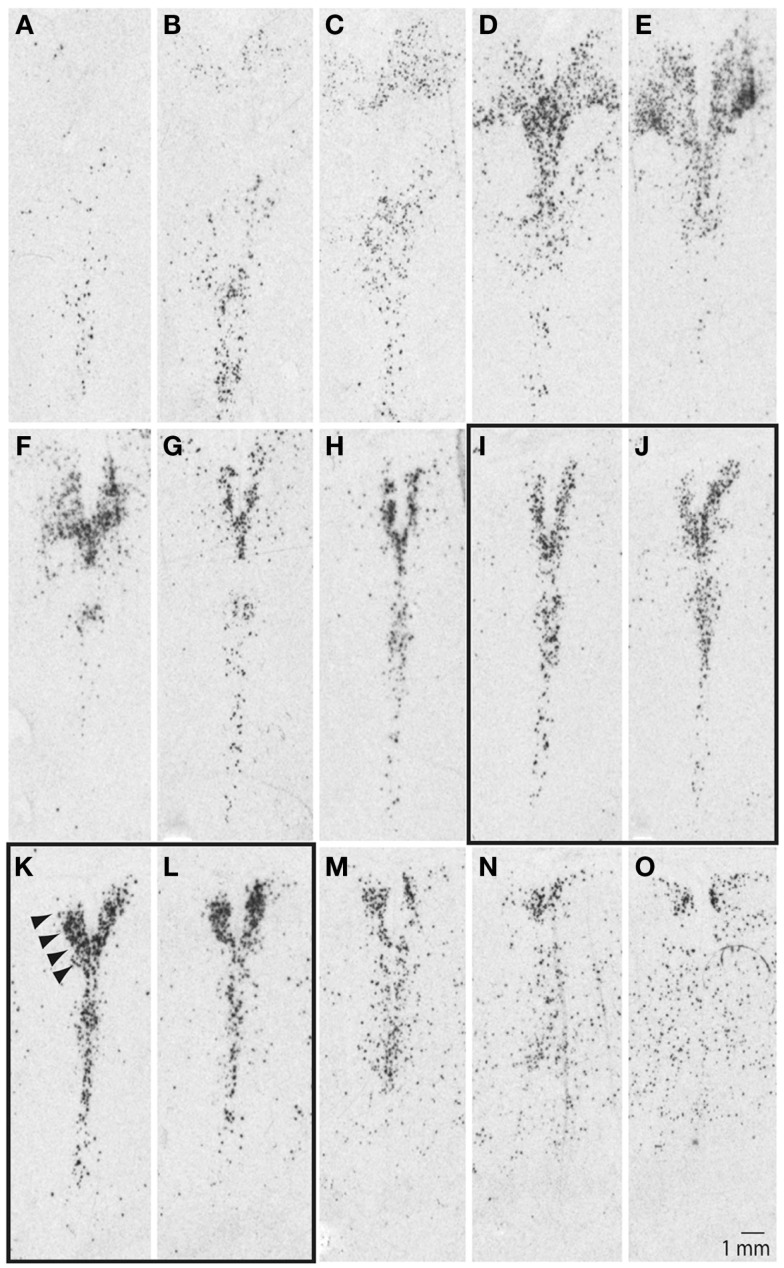
**Serotonin transporter (SERT) mRNA defines serotonergic cell groups in the brainstem**. Sections are arranged from anterior **(A)** to posterior **(O)** pole of the raphe nuclei in 500 μm intervals. Arrowheads in **(K)** identify location of the dorsal raphe. Note differences in its shape, as well as distribution and density of the signal within the: rostral **(A–C)**, middle-rostral **(D–F)**, middle-caudal **(G–L)**, and caudal **(M–O)** subdivisions of the dorsal raphe (see text for additional details). LCM samples were collected from the middle-caudal region of the dorsal raphe, panels **(I–L)**. Sections were processed for radioactive *in situ* hybridization using a riboprobe based on NCBI nucleotide accession #NM 001045.2, pos. 705–1789.

Our initial studies examined ISH autoradiograms to delineate the organization of the DR and the neighboring 5-HT cell groups in the human brainstem. Figure 1 illustrates a series SERT autoradiograms from a representative control subject, with images arranged from the anterior (Figure 1A) to the posterior (Figure 1O) pole of the DR in 500 μm increments between adjacent panels. At its anterior pole, the DR was barely noticeable and was defined by loosely packed and scattered SERT signal (Figures 1A–C); moving more posteriorly, the DR was defined by fan-like shape with loosely packed horns located dorsolaterally to its vertically oriented midline division (Figures 1D–F). At its middle and middle-caudal levels (Figures 1G–L), the dorsolateral horns narrowed and increased their density of hybridization signal, and the overall shape of the DR resembled the letter V as indicated by arrowheads in Figure 1K. At the more caudal levels, the DR became loosely packed with ill-defined borders and was contiguous with dispersed 5-HT cell groups located ventrally (Figures 1M–O).

These rostro-caudal differences in the organization of the DR correspond with functional and connectional differences (Abrams et al., 2004; Lowry et al., 2008). Tract-tracing studies in animal models have demonstrated that the caudal portion of the DR receives strong afferent inputs from several forebrain limbic regions, including medial prefrontal cortex, habenula, and the hypothalamus, while giving rise to projections to the hippocampus and lateral septum (Wyss et al., 1979; Lee et al., 2003; Waselus et al., 2006). Previous post-mortem human brain studies have also demonstrated more robust gene expression differences within the middle-caudal DR, as opposed to its more rostral subdivisions, in depressed suicide individuals (Bach-Mizrachi et al., 2006, 2008). Based on these observations we collected our samples from the middle-caudal level of the DR (Figures 1I–L).

### RNA quality

Quality of extracted RNA was determined by analyzing electropherograms obtained from 2100 BioAnalyzer. Previous work has demonstrated that samples with ≤65% of their signal (quantified by area under the curve) before the 18S rRNA peak and with ≥4% of the signal within the 28S rRNA peak are compatible with gene expression profiling analyses (Schoor et al., 2003; Bernard et al., 2009, 2011). In our control samples we observed 48.5 ± 3.27% (mean ± SEM; 95% CI: 46.0–50.9%) of signal <18S area of electropherogram, and 7.2 ± 1.5% (95% CI: 4.4–10.1%) within the 28S peak. In the MDD samples these measures were: 50.5 ± 1.6% (95% CI: 48.9–52.1%) and 7.5 ± 0.7% (95% CI: 5.6–9.4), respectively. Since group differences were not significant (*p* > 0.05) and observed values were all well above inclusion thresholds, these data indicate that extracted RNA was compatible with downstream gene expression profiling and qPCR quantification.

### Overall microarray results

Overall expression intensity for the microarray data from RMA log_2_ values was 6.57 ± 0.01 in the C group and 6.58 ± 0.01 in the MDD group. Raw detection call rates calculated from MAS5CALLS output were 49.0 ± 1.4% for the C group and 47.9 ± 1.5% for the MDD group. We found no significant differences in RMA intensity values or MAS5CALLS detection call rates (*p* > 0.05).

**Figure 2:**
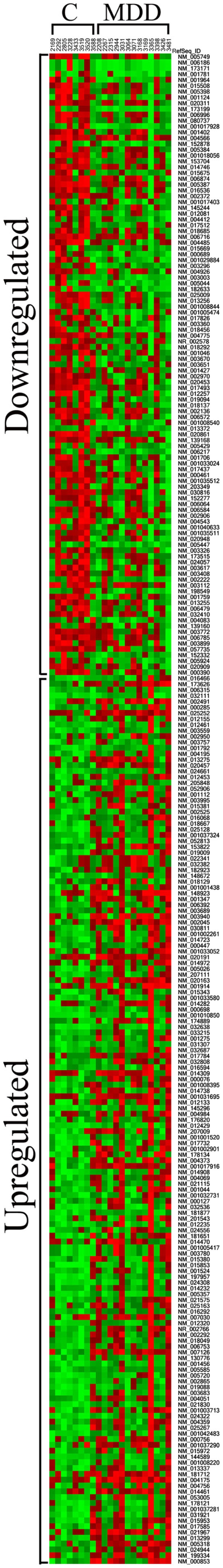
**Heatmap of differentially expressed genes included in functional pathways analysis (see text for more details)**. It was generated by using the method of King et al. (2005) and publicly available software at http://ashleylab.stanford.edu/tools_scripts.html.

The average Pearson correlation among all of the samples was 0.95 ± 0.007. One of the samples (subject 3365) had an average correlation of 0.83 with the other samples. However, removing this sample from our analyses, did not significantly impact the largest changes in gene expression that we detected (see Tables 3 and 4). Heat map of differentially expressed genes included in functional pathways analysis (see below) revealed a clear separation between C and MDD samples (Figure 2).

**Table 3:** **Functional groups that showed significant alterations in gene expression**.

**N-glycan biosynthesis**	**Intracellular signaling**
B4GALT6 ↓	ANLN ↓
MAN2A1 ↓	***PPP1R3C*** ↓
B4GALT2 ↑	ITPR1 ↓
DOLK ↑	GNG4 ↓
RPN1 ↑	GNA13 ↓
	PRKAG2 ↓
**Metabolism**	RDX ↓
ALDH1A1 ↓	SAT1 ↓
SDHC ↓	PIP4K2B ↑
BDH1 ↑	PARD6A ↑
DGKQ ↑	CDH2 ↑
LIPE ↑	TOLLIP ↑
	VAMP2 ↑
**Axonal guidance signaling**	NPR2 ↑
CXCR4 ↓	
ARHGEF7 ↓	**Transcription regulators**
VEGFC ↓	***EGR1*** ↓
SEMA3G ↑	***TOB1*** ↓
KLC1 ↑	***NR4A2*** ↓
RND1 ↑	***NR4A3*** ↓
ARPC1B ↑	SMAD6 ↑
LINGOl ↑	
PIK3CD ↑	**NRF2-mediated oxidative stress response**
	MAFF ↓
**Mitochondrial dysfunction**	AKR7A2 ↑
F1S1 ↑	***GSTT1*** ↑
NDUFB3 ↑	VCP ↑
COX6A1 ↑	CYP4Z1 ↑
PRDX5 ↑	CDC34 ↑
PSEN2 ↑	
	**Cell cycle regulation**
	HNRNPA1 ↓
	CCNE2 ↓
	CCND2 ↓.
	TINF2 ↑
	MAX ↑

*Using Ingenuity Pathway Analysis we identified a total of 21 canonical pathways that exhibited significant alterations in expression (*p* < 0.05). These pathways belonged to eight distinct functional groups (bold headings); within each functional group genes that were significantly altered in the MDD group as compared to the C group are listed with arrows indicating upregulation or downregulation in expression. Bold italics indicate genes that were among those that showed the largest changes in expression (see Table 4)*.

**Table 4:** **Results of microarray and qPCR experiments**.

Ref seq ID	Gene name	Symbol	Gene expression Microarray			qPCR		Array-qPCR	
			Log 2 mean intensity	*p*-Value	*F*-change	*p*-Value	*F*-change	*p*-Value	*r*
Genes (log2 intensity > 4; *p* < 0.05; fold-change > 1.4)		
NM_005749	Transducer of ERBB2, 1	TOB1	10.1	**0.034**	−1.95	**0.021**	−1.67	<0.0001	0.90
NM_173173	Nuclear receptor subfamily 4, group A, member 2	NR4A2	6.3	**0.007**	−1.87	**0.032**	−3.57	<0.01	0.63
NM_001964	Early growth response 1	EGR1	8.8	**0.023**	−1.71	**0.046**	−2.96	<0.01	0.59
NM_001781	CD69 molecule	CD69	5.3	**0.036**	−1.72	*0.076*	−2.68	<0.05	0.51
NM_015508	TCDD-inducible poly(ADP−ribose) polymerase	TIPARP	9.0	**0.012**	−1.60	**0.038**	−2.26	<0.0001	0.93
NM_001124	Adrenomedullin	ADM	9.3	**0.008**	−1.59	**0.025**	−2.81	<0.0001	0.70
NM_005398	Protein phosphatase 1, regulatory (inhibitor) subunit 3C	PPP1R3C	8.9	**0.010**	−1.59	*0.061*	−2.03	<0.0001	0.95
NM_020311	CXCR7 chemokine (C−X−C motif) receptor 7	CXCR7	8.7	**0.049**	−1.57	*0.098*	−1.67	<0.0001	0.90
NM_173199	Nuclear receptor subfamily 4, group A, member 3 (NR4A3)	NR4A3	5.6	**0.001**	−1.54	0.127	−1.51	0.46	0.17
NM_006996	Solute carrier family 19 (thiamine transporter), member 2	SLC19A2	7.0	**0.041**	−1.53	0.125	−1.58	<0.0001	0.87
NM_001402	Eukaryotic translation elongation factor 1 alpha 1	EEF1A1	8.4	**0.016**	−1.42	0.117	−2.36	<0.0001	0.58
NM_001017928	Coiled-coil domain containing 58	CCDC58	5.5	**0.003**	−1.48				
NM 080737	Svnaptotagmin-like 4	SYTL4	6.3	**0.043**	−1.49				
NM_153010	CHST9 antisense RNA 1 (non-protein coding)	CHST9-AS1	6.7	**0.017**	−1.59				
NM_024854	Pyridine nucleotide-disulphide oxidoreductase domain 1	PYROXD1	9.6	**0.022**	1.56				
NM 000853	Glutathione S-transferase theta 1	GSTT1	5.6	**0.014**	1.43				

**F*-change indicates fold change in the MDD group as compared to the C group. Significant (*p* < 0.05) changes are indicated in bold; those that exhibited a trend toward significance (*p* < 0.1) are italicized. Correlations between microarray and qPCR results are also presented; *r*, Pearson’s *r* correlation coefficient*.

### Functional pathways

To evaluate differential expression we used the MAS5CALLS algorithm, which computes through selective match/mismatch ratio, the certainty with which a transcript is detected on a microarray chip. Genes that had log_2_ RMA mean intensity values ≥4.0, exhibited MAS5CALLS detection call rates ≥50% in C or MDD group, were upregulated or downregulated by ≥1.1-fold at *p* < 0.05 level were included. Using these criteria, a total of 292 genes were found to be differentially expressed between C and MDD samples, and of these, 126 genes were downregulated and 166 were upregulated (see Supplementary Materials). We utilized Ingenuity Pathway Analysis^5^ to identify functional pathways that are impacted by these expression changes. A total of 21 cannonical pathways were identified as significantly involved (*p* < 0.05); these pathways were grouped into 8 functional categories: *n*-glycan biosynthesis, metabolism, axonal guidance signaling, mitochondrial dysfunction, intracellular signaling, transcription regulators, NRF2-mediated oxidative stress response, and cell cycle regulation (Table 3).

### Transcription regulators and downstream targets

As the next step, we used an unbiased analysis approach to detect genes that exhibited the largest differences in expression between C and MDD samples. Microarray data were filtered for genes that were identified as detected by MAS5CALLS algorithm on at least 50% of arrays in either group, had log_2_ RMA mean intensity values ≥4.0, and showed ≥1.4-fold changes in expression at *p* < 0.05. This approach revealed alterations in the expression of 16 transcripts in MDD versus C. Expression of 13 of these genes was downregulated, including: TOB1, NR4A2, EGR1, CD69, TIPARP, ADM, PPP1R3C, CXCR7, NR4A3, SLC19A2, EEF1A1, CCDC58, and SYTL4 (Table 4). Expression of two genes, GSTT1 and PYROXD1, was upregulated, while that of a non-protein coding RNA CHST9-AS1 was downregulated in the MDD group (Table 4).

Examination of the top genes within the framework of pathway analysis revealed that three functional groups – (1) Intracellular Signaling, (2) Transcription Regulators, and (3) NRF2-Mediated Oxidative Stress Response, contained genes with the largest (≥1.40-fold change) changes in expression. Of these, the Transcription Regulators functional group contained the greatest number of such genes, which included: EGR1, TOB1, NR4A2, and NR4A3 (Tables 3 and 4).

Confirmation of expression changes by qPCR was conducted on samples from all subjects and included 11 transcripts. The changes in expression detected by both microarrays and by qPCR were always in the same direction (Table 4). We observed a significant (*p* < 0.05) correlation between microarray and qPCR results for all genes, with the exception of NR4A3 (Table 4). Changes in the expression of five transcripts (TOB1, NR4A2, EGR1, TIPARP, and ADM) reached statistical significance (*p* ≤ 0.05) as detected by qPCR, whereas changes in the expression of three other genes (CD69, PPP1R3C, and CXCR7) exhibited a trend toward significance (*p* ≤ 0.1; Table 4).

**Figure 3:**
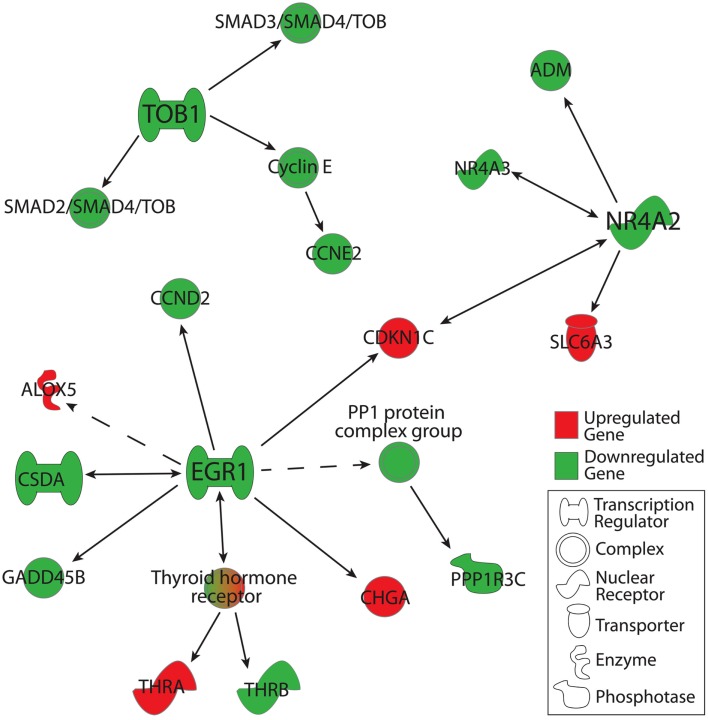
**Downstream gene targets of TOB1, EGR1, and NR4A2 that were altered in their expression in the MDD group as compared to the C group**. Genes were included in this analysis if: (1) they were detected in ≥50% of arrays in either the MDD or C group, (2) their changes in expression were ≥1.1- or ≤−1.1-fold in magnitude, and (3) associated *p*-values < 0.05. Gene-gene interactions were analyzed using Ingenuity Pathway Analysis knowledge database. Marked in red are gene products that were upregulated, while those in green were downregulated.

Using IPA functional networks feature, we extended our analysis to downstream targets of TOB1, NR4A2, and EGR1. Genes were included in this analysis if they were detected in ≥50% of C or MDD microarrays and were altered in their expression by 1.1-fold or greater at the level of *p* < 0.05. In the case of TOB1, this analysis revealed a downregulation of SMAD2-SMAD4-TOB and SMAD3-SMAD4-TOB transcriptional regulation complexes along with the downregulation of CCNE2, a cell cycle regulator that belongs to the Cyclin E complex (Figure 3). Expression of multiple genes was downregulated downstream of EGR1, including that of: CCND2, CSDA (which has a reciprocal relationship with EGR1), GADD45B, PPP1R3C, and THRB. Additionally, expression of CHGA, THRA, ALOX5, and CDKN1C was upregulated downstream from EGR1. In the case of NR4A2, its downstream targets of ADM and NR4A3 were downregulated, while CDKN1C and SLC6A3 were upregulated. Interestingly, NR4A3 exhibits a reciprocal relationship with NR4A2, while CDKN1C expression is regulated by both NR4A2 and EGR1 (Figure 3).

**Figure 4:**
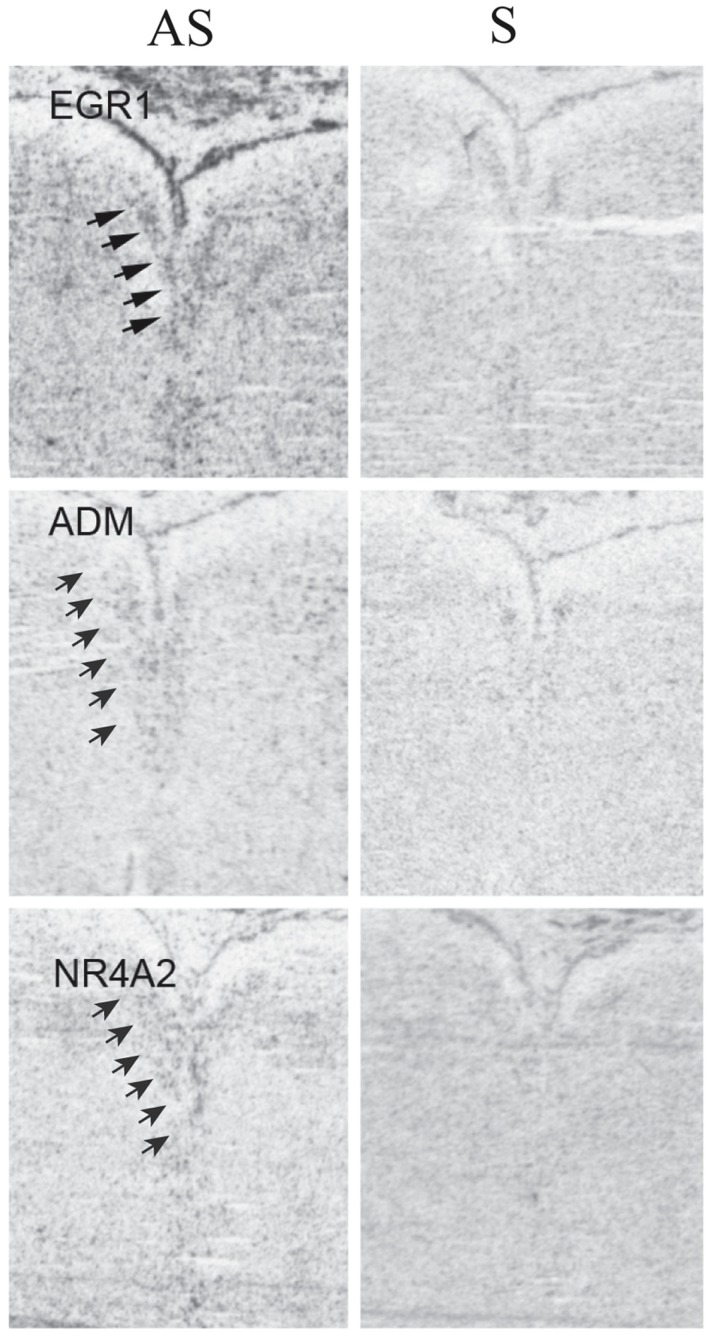
***In situ* hybridizations for EGR1, ADM, and NR4A2**. Tissue was processed for radioactive ISHs as previously described (Lopez-Figueroa et al., 2004; Bernard et al., 2009), using the following probes: EGR1, accession # NM_001964, pos. 857–1495; ADM, accession # NM_001124, pos. 701–1349; NR4A2, accession # NM_NM 173173, pos. 344–669. Binding with antisense (left column) and sense (right column) riboprobes is illustrated to compare specific and non-specific binding. Arrows indicate location of the dorsal raphe; compare to signal in Figures 1I–L. Note the presence of specific signal that is enriched within the DR in the antisense images, but is absent in the sense ones.

To determine the pattern of expression within the DR of EGR1, NR4A2, and ADM (a downstream target of NR4A2), we performed ISH in three of the control subjects on tissue sections that contained the middle-caudal DR (Figure 4). These genes were widely expressed at relatively low levels, as is true of other transcriptional regulators (Kerman et al., 2012). These ISH data suggest that EGR1, NR4A2, and ADM are expressed within a variety of cell types, but are enriched in their expression within the DR (Figure 4). Based on this hybridization pattern, it is likely that these genes impact a wide variety of cellular functions and neuronal circuits.

### Impact of medication

**Figure 5:**
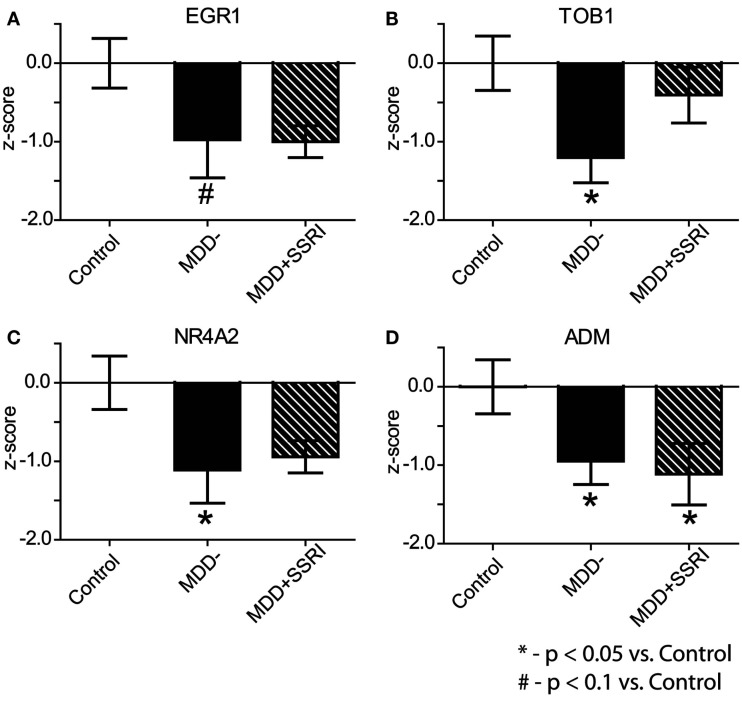
**Effect of SSRI treatment on gene expression**. Within subject microarray and qPCR *z*-scores were combined for each gene: EGR1 **(A)**, TOB1 **(B)**, NR4A2 **(C)**, and ADM **(D)**. Data are shown as Mean ± SEM *z*-scores for: Contol subjects (*n* = 8), MDD subjects not treated with SSRIs (MDD−; *n* = 8) at the time of death, and MDD subjects treated with SSRIs (MDD + SSRI; *n* = 5) at the time of death (see text for more details).

To assess potential medication effects, we examined expression levels of EGR1, TOB1, NR4A2, and ADM in MDD patients who were prescribed SSRIs at the time of death (MDD + SSRI) and in those who were not (MDD−). Because microarrays and qPCR are complementary measures of gene expression we used a meta-analytic approach of *z*-score normalization to combine microarray and qPCR results (Jain et al., 2005; Guilloux et al., 2011). Individual *z*-scores were calculated by normalizing differences between each data point and the mean of the C group to the standard deviation of the C group. Within subject microarray and qPCR *z*-scores were averaged for each gene, and this average *z*-score was then used for group comparisons. This analysis revealed significant (*p* < 0.05) downregulation in the expression of TOB1, NR4A2, and ADM in MDD-subjects, and a trend (*p* < 0.1) for downregulation for EGR1, as compared to C subjects (Figure 5). In the MDD + SSRI group, we observed significant (*p* < 0.05) downregulation in the expression of ADM, and a trend for downregulation in EGR1 (*p* = 0.1) and NR4A2 (*p* = 0.1), as compared to the C group.

## Discussion

This study examined DR in post-mortem brains from patients diagnosed with MDD and from psychiatrically normal comparison subjects. We utilized the ISH-guided LCM approach to collect DR samples, because our previous studies validated this approach in gene expression studies in postmortem human brain tissue (Bernard et al., 2009, 2011). This method combines a high level of anatomical resolution with more precise tissue sampling, which enriches specific mRNAs and improves sensitivity and the dynamic range of microarray-based gene expression profiling (Bernard et al., 2009). We also chose to look at the entire region of DR within each section rather than capturing individual neurons, because previous studies indicate the importance of the entire cellular milieu of the DR, including neurons of different neurochemical content (i.e., 5-HT and non-5-HT containing; Lemos et al., 2006). Other studies have also highlighted the importance of glia and glial-neuronal interaction in normal brain function, as well as in MDD, in multiple cortical and subcortical brain regions (Rajkowska and Miguel-Hidalgo, 2007; Bernard et al., 2011; Parpura et al., 2012). Our microarray data suggest that a number of functional gene networks are altered in the DR in the MDD brain. These include: biosynthesis and metabolism, axonal guidance, mitochondrial dysfunction and oxidative stress, cell cycle regulation, and intracellular signaling and transcriptional regulation. Of these functional groups, genes with the largest changes in expression belonged to the transcriptional regulation group, and subsequent qPCR validation confirmed the downregulation in the expression of EGR1, TOB1, and NR4A2. These changes did not appear to be due to medication effects, and functional pathway analysis of the microarray data revealed alterations in the expression of several downstream targets of these transcriptional regulators. Our preliminary anatomical studies suggest that EGR1 and NR4A2, as well as ADM (a downstream target of NR4A2 that was also significantly downregulated in MDD), are widely expressed, but are enriched in their expression within the DR. These observations suggest transcriptional alterations within multiple cell types in the DR, which may have diverse functional consequences on its function.

### Limitations

As in all post-mortem human brain investigations, our study has several methodological limitations. Most importantly, one needs to be aware that our observations are correlational, and thus we cannot know from our data whether these molecular alterations contribute to the emergence of MDD or are its consequence. Nonetheless, our data are consistent with other studies that have implicated EGR1, NR4A2, and TOB1 in a number of functional processes that are dysregulated in neuropsychiatric disorders (see below).

We focused on gene expression at the middle-caudal portion of the DR, because 5-HT neurons within the rostral DR are more likely to project to motor targets, while those located more caudally are enriched in their projections to the forebrain limbic targets, including hippocampus and lateral septum (Wyss et al., 1979; Waselus et al., 2006). These limbic nuclei play an important role in reward and memory function, which are impaired in MDD. Previous post-mortem studies also reported gene expression alterations in the DR of depressed suicides, with the largest differences within its middle-caudal portion (Bach-Mizrachi et al., 2006, 2008). Taken together, these studies suggest that the middle-caudal DR plays an important role in affective regulation and is specifically impacted in MDD. Future work will be required to determine whether gene expression changes that we observed in this DR subregion extend to its more rostral subdivisions.

Subjects included in our study were severely depressed at the time of death. Nearly half of them committed suicide, which was likely related to their depression. Therefore, it may be that our observed gene expression changes are not present in mildly or moderately depressed individuals. In addition, in most post-mortem studies of neuropsychiatric diseases it is very difficult to determine whether observed effects are due to the illness itself, or due to the medications used to treat the disease. To address this issue we parsed MDD subjects into those who were and into those who were not prescribed SSRIs at the time of death. This analysis demonstrated significant downregulation in the expression of TOB1, NR4A2, and ADM, and a trend toward significance for EGR1, in the latter group relative to the controls. Though we cannot rule out potential impact of other drugs, these results suggest that our observations are not due to SSRIs, the most common medications used to treat MDD. Nonetheless, the observed alterations in gene expression need to be replicated in an independent cohort of subjects to increase confidence in our results.

We designed the present study to examine gene expression alterations within the entire cellular milieu of the middle-caudal DR. The advantage of this approach is that we were able to: (1) target the portion of the DR that regulates limbic functions, (2) examine gene expression alterations within multiple cell types, and (3) identify functional pathways most strongly impacted by MDD. However, the drawback of this approach is that it lacks cellular resolution and we cannot know whether these gene expression changes impact a variety of cell types within the DR, or if they are restricted to a particular functional class. Future studies that utilize single cell analyses will be required to address this issue.

### Functional pathways alterations

Pathway analysis of the microarray data revealed dysregulation in the DR of MDD patients within several functional gene groups, including those that regulate: biosynthesis, metabolism, axon guidance, mitochondrial function, intracellular signaling, transcription, oxidative stress response, and the cell cycle. These data suggest that a defined set of cellular functions are dysregulated in the DR in MDD, including: (1) intracellular stress and energy balance, (2) intracellular signaling and transcriptional regulation, and (3) cell proliferation and connectivity.

Previous studies have implicated these, or related, functional groups in the pathophysiology of mood disorders in other brain regions. For example, decreased cell proliferation may contribute to the decrease in the numbers of glial cells and glial markers within cortical and subcortical brain regions in the depressed brain (Rajkowska, 2003; Rajkowska and Miguel-Hidalgo, 2007; Bernard et al., 2011). Likewise, increased hippocampal neurogenesis has been proposed to underlie the mechanism of antidepressant action (Perera et al., 2007; Boldrini et al., 2009). Alterations in the expression of cell connectivity markers are consistent with imaging reports demonstrating alterations in resting-state connectivity among several limbic brain regions in MDD (Fingelkurts et al., 2007; Greicius et al., 2007). Mitochondrial dysfunction and impaired intracellular stress response have also been implicated in the emergence of bipolar disorder and depression (Rezin et al., 2009). Taken together with our data, these observations suggest that MDD is associated with the dysregulation of a set of functional pathways across multiple brain regions. Future work will be required to determine how these alterations impact specific cell types and circuits, and how these functional changes mediate clinical manifestations of mood disorders.

### Transcriptional dysregulation in the DR

Cellular transcription is a process that is tightly controlled by DNA-binding factors that regulate a variety of process, such as neurodevelopment, adaption to environmental cues, intracellular signaling, cell cycle control, and others (Collins et al., 1995; Cho et al., 2001; Lonze and Ginty, 2002). Thus, in the intracellular signaling chain, transcriptional regulators are at the cusp between the effects of an external stimulus and its impact on cellular function. Altered transcriptional control can result in dysregulated expression of a host of downstream genes, and such changes have been observed in cancer, autoimmune diseases, diabetes as well as in neuropsychiatric disorders (Abraham and Kroeger, 1999; Black et al., 2001; Habener et al., 2005; Monteggia and Kavalali, 2009; Robison and Nestler, 2011). Many of these studies report global transcriptional alterations, but local circuitries may contain yet undetected defects in transcriptional control. Indeed, upregulation in the expression of NUDR and REST, both of which are 5-HT-related transcriptional regulators, have been recently reported within serotonergic neurons in the DR (Goswami et al., 2010). Interestingly, these changes were sex-dependent and were only apparent in female subjects. Here we report that among the DR transcripts that exhibited the most significant and largest expression differences in MDD, there are three transcription factors: TOB1, NR4A2, and EGR1.

Because these genes are transcriptional regulators, we used functional pathway analysis to identify changes in expression of their downstream targets. This approach revealed alterations in the expression of several downstream targets in our microarray data set. These included other transcriptional regulators: NR4A3, THRA, THRB, and CSDA. In addition to these direct transcriptional regulators, we also observed a downregulation in the expression of GADD45B, a downstream target of EGR1 and an epigenetic regulator that mediates activity-dependent DNA demethylation (Ma et al., 2009). These observations suggest alterations in transcriptional regulation via changes in the expression of factors that bind directly to the genome, as well as those that mediate epigenetic regulation of gene expression.

Analysis of downstream targets also revealed downregulation in the expression of CCND2 and CCNE2, downstream targets of EGR1 and TOB1, respectively. Both of these genes are part of the cell cycle regulation group, which we observed as significantly altered using functional pathway analysis (Table 3). We also detected a downregulation in the expression of PPP1R3C, downstream of EGR1, and that of ADM, downstream of NR4A2, both of which were among the genes that showed that largest changes in expression (Table 4). ADM is a widely expressed peptide that exerts its actions via a G protein coupled receptor, which when activated triggers a second messenger cascade that regulates gene transcription (Cuttitta et al., 2002). Altered expression of ADM has been previously correlated with mood disorders, while its knockout in mice leads to heightened anxiety-like behavior (Savas et al., 2002; Fernandez et al., 2008; Huang et al., 2010). In addition, a recent study has identified NR4A2 as a regulator of EGR1 expression, raising the possibility that these transcriptional regulators also directly interact with each other (Johnson et al., 2011).

Previous studies have implicated alterations in the expression or function of EGR1, NR4A2, and TOB1 in brain processes impacted by MDD. For example, TOB1 expression is altered in the frontal cortex of patients with bipolar disorder (Bezchlibnyk et al., 2001), while molecular interference with the TOB1 protein suppresses long-term potentiation and impairs spatial learning and memory in rats (Jin et al., 2005). Previous work has demonstrated that TOB-interacting proteins play a role in neuronal differentiation, suggesting that TOB1 is expressed in neurons (Berthet et al., 2002). TOB1 plays a fundamental role in cell proliferation and growth (Maekawa et al., 2002), and it is feasible that its dysfunction may lead to morphological changes observed in the DR of MDD patients (Baumann et al., 2002).

Studies focused on NR4A2 have demonstrated its importance in the genesis of dopaminergic neurons (Ojeda et al., 2003; Galleguillos et al., 2010). In addition to the decrease in NR4A2 expression, functional pathway analysis of our data also revealed an upregulation in the expression of the dopamine transporter (SLC6A3; Figure 3). DR contains a large number of catecholaminergic neurons that give rise to ascending projections to nucleus accumbens, prefrontal cortex, and the lateral septum, key areas in the regulation of affect and reward (Stratford and Wirtshafter, 1990; Michelsen et al., 2007). Taken together, these observations suggest that functional alterations of ascending catecholamine circuits from the DR may contribute to the etiology of MDD. Consistent with this notion is the observation that mice with reduced NRA2 exhibit alterations in forebrain levels of dopamine and 5-HT along with increased immobility on the forced swim test, a measure of learned helplessness and a core feature of MDD (Rojas et al., 2010).

EGR1 is a transcriptional switch that regulates a number of diverse gene targets. It is an immediate-early gene that gets activated in response to a variety of stimuli, including drug administration that leads to 5-HT release (Humblot et al., 1998). Previous work indicates that it is strongly expressed in neurons in the adult brain, and that its levels are altered by neurotransmitter release and neuronal activation (Knapska and Kaczmarek, 2004). It can also exert long-lasting changes in gene expression and subsequent protein synthesis that mediate synaptic plasticity. EGR1 has been implicated in mediating a variety of behaviors that are dysregulated in MDD, including learning and memory, fear conditioning, drug addiction, and social interaction (Cole et al., 1989; Ressler et al., 2002; Malkani et al., 2004; Valjent et al., 2006; Stack et al., 2010). Extensive evidence also implicates 5-HT signaling in mediating neuroplasticity, such as functional plasticity in the cortex following sensory deprivation (Maya Vetencourt et al., 2008; Jitsuki et al., 2011). These observations raise the possibility that altered EGR1 expression in the DR may lead to disruption of 5-HT-mediated plasticity within the limbic forebrain regions.

A recent report has documented a decrease in the cross-sectional area of the DR in MDD (Matthews and Harrison, 2012). This observation may be due to a variety of factors, including decreased numbers of neurons or glia, or decreased density of synaptic inputs or diminished dendritic branching of DR neurons. Given that TOB1 and NRA2 play an important role in neuronal differentiation, it is feasible that alterations in their expression may contribute to morphological alterations within the DR associated with MDD.

## Conclusion

Transcriptional regulators mediate a number of brain functions and behaviors that are dysregulated in MDD, including learning and memory, fear consolidation, and cognitive function (Morris et al., 1988; Ressler et al., 2002; Malkani et al., 2004). Transcriptional dysregulation has also been previously implicated in the etiology of diverse neuropsychiatric disorders such as Rett syndrome, addiction, schizophrenia, bipolar disorder, and MDD (Law et al., 2006; Monteggia and Kavalali, 2009; Goswami et al., 2010; Soria et al., 2010; Robison and Nestler, 2011). Because of its key role in central 5-HT neurotransmission and extensive evidence for its functional and structural alterations in mood disorders, along with similar alterations in rodent models of depression, the DR likely plays a key role in the pathophysiology of MDD. Our data point to the dysregulation of multiple transcriptional regulators in the DR, including: (1) transcription factors that directly bind to the genome in response to upstream gene activation or environmental cues (i.e., EGR1, TOB1, and CSDA), (2) ligand-activated nuclear receptors that regulate transcription via their genomic actions (NR4A2, NR4A3, THRA and THRB), (3) an epigenetic regulator (GAAD45B), and (4) a peptide that regulates transcription via its binding to a G protein coupled receptor and downstream activation of intracellular signaling (ADM). These observations suggest alterations in the function of a host of downstream targets, which may lead to the dysregulation of multiple cellular functions that contribute to the pathophysiology of MDD. Future studies will require single cell analyses in the DR to determine potential impact of these changes on its cellular functions and related circuits. One approach would be to use data from previous studies that used microarrays to fingerprint individual classes of cells in the brain (Ponomarev et al., 2006). However, since such data are not available for the human brainstem, studies utilizing single cell LCM in the postmortem human brain will be required (Goswami et al., 2010; Ordway et al., 2012).

## Disclaimer

This work was performed as part of the Pritzker Neuropsychiatric Disorders Research Consortium (http://www.pritzkerneuropsych.org/), a large multi-site effort dedicated to uncovering neurobiological underpinnings of neuropsychiatric diseases. The data presented are part of a much larger data set, which is still being mined by the Consortium for other aspects of gene expression alterations from the major depressive cohort. The raw microarray data are not being released as part of this paper. However, the Consortium is planning a large release of the raw microarray data from multiple brain regions in the postmortem human tissue. A list of differentially expressed genes detected in this study is included in Supplemental Materials.

## Conflict of Interest Statement

The authors declare that the research was conducted in the absence of any commercial or financial relationships that could be construed as a potential conflict of interest.

## Supplementary Material

The Supplementary Material for this article can be found online at http://www.frontiersin.org/Neurogenomics/10.3389/fnins.2012.00135/abstract

## Footnotes

^1^http://www.kodak.com

^2^http://brainarray.mbni.med.umich.edu/Brainarray/Database/CustomCDF/genomic_curated_CDF.asp

^3^http://www.ncbi.nlm.nih.gov/entrez/query.fcgi?CMD = search&DB = gene

^4^http://www.bioinfo.rpi.edu/applications/mfold/

^5^http://www.ingenuity.com/products/pathways_analysis.html
